# Editorial: Navigating child injury prevention and safety promotion: strategies, evidence, and sustainable transformations

**DOI:** 10.3389/fpubh.2026.1992274

**Published:** 2026-10-26

**Authors:** Ye Jin, Li Li, Julie Brown, Pengpeng Ye

**Affiliations:** 1 National Center for Chronic and Noncommunicable Disease Control and Prevention, Chinese Center for Disease Control and Prevention, Beijing, China; 2 Department of Epidemiology and Health Statistics, Xiangya School of Public Health, Central South University, Changsha, Hunan, China; 3 The George Institute for Global Health, University of New South Wales, Sydney, NSW, Australia

**Keywords:** child injury, health equity, injury prevention, safety promotion, social determinants

When we first conceived this Research Topic, our aim was straightforward: to bring together evidence on what works, for whom, and under what conditions, in preventing injuries among children and adolescents. The need for such a Research Topic was clear. Each year, close to one million children and adolescents die from injuries worldwide, and with a disproportionate burden in low- and middle-income countries (LMICs). Non-fatal injuries add a further, often invisible, layer of burden—disrupted schooling, impaired physical and cognitive development, and families pushed deeper into poverty (1, 2). Childhood injury mortality has declined substantially in many high-income countries over recent decades, alongside advances in injury surveillance, safer environments and products, and evidence-based legislation and enforcement (1). Yet in LMICs, fragmented data systems, constrained resources, and widening equity gaps continue to leave the most vulnerable children exposed (3, 4). Children with disabilities, left-behind children in rural communities, ethnic minorities, and those living in poverty carry a disproportionate share of this burden, yet their specific risks remain understudied and underaddressed. Without tackling injury as a core, preventable cause of child morbidity and mortality, the Sustainable Development Goal 3.2 target of ending preventable deaths among newborns and children under five will remain a challenge (5, 6).

This Research Topic brings together 26 contributions, for example, original research, systematic reviews, qualitative inquiries, and methodological innovations, from Africa, Asia, the Americas, and Europe. Several studies advance our understanding of child injury epidemiology. Others evaluate the effectiveness and scalability of prevention strategies, or examine the structural barriers that slow policy translation. A subset explores newer methodologies, including artificial intelligence, spatial modeling, and participatory approaches. Taken together, these papers prompt a question that has long occupied us as editors: how do we move beyond simply documenting disparities, and toward producing solutions that are actionable, equitable, and adapted to the contexts in which children actually live?

The geographic spread of contributions is wide, though not evenly distributed. Sixteen studies come from China, covering twelve provinces and municipalities from eastern cities to western mountain regions. The remaining 10 cover Bangladesh, Nepal, Saudi Arabia, Poland, Germany, the United States, and French Guiana, spanning high-income and low-income settings. Drowning and road traffic injury receive the most attention, but the Topic also includes work on falls, burns, poisoning, sports injuries, emotional bullying, concussion, and multiple fractures. Methodologically, observational designs predominate—retrospective cohort studies, cross-sectional surveys, and time-series analyses—supplying epidemiological intelligence across multiple scales. Intervention studies, systematic reviews, and qualitative inquiries are fewer in number, but they add essential evidence on effect evaluation and implementation. Nearly three-quarters of the studies are situated in LMICs, which aligns the evidence base with the geographic distribution of the global burden. The study populations center heavily on children under five, consistent with their particular vulnerability to injury. Adolescents and children with disabilities are less represented—a gap that should be considered when interpreting the scope and generalizability of the evidence presented in this Research Topic.

## Mapping the burden: surveillance, trends, and population-level patterns

Prevention depends on surveillance that can look beyond headline counts to identify where declines are real, where risk remains concentrated, and which subgroups continue to be missed. Zhang et al. offer a 32-year retrospective analysis of injury-related mortality among children and adolescents in central Shanghai. They document significant declines in traffic injuries, drowning, falls, and accidental poisoning, trends that track with the rollout of traffic safety policies, school zone improvements, and product safety regulations. Yet suicide among older adolescents persists, a finding that signals an unmet need for mental health infrastructure and school-based crisis intervention. Wang et al. report a representative cross-sectional survey from Henan Province, identifying boys, day students, and children under grandparental care as high-risk subgroups and documenting substantial health and economic consequences. These two studies make a practical point—surveillance must capture the full spectrum of burden if it is to guide resource allocation and targeted action.

## Drowning: from perception gaps to clinical prediction

Drowning is well-represented in this Topic, reflecting its role as a leading cause of child mortality globally. Alam et al. describe a sharp perception-action paradox in rural Bangladesh. Over 90% of caregivers believe drowning is preventable, yet fewer than 6% teach their children how to respond to a witnessed drowning, and over 84% lack rescue knowledge. These gaps persist despite government plans to expand community crèches—supervised childcare sites intended to keep young children away from water hazards while parents work. The findings suggest that such expansion is necessary but not sufficient on its own: without safety audits, trained supervision, and consistent quality standards, even well-intentioned programs may not reach their preventive potential. Zhong et al. observe a similar paradox among Chinese adolescents: drowning prevention knowledge is high, yet risky water behaviors continue, especially among males and boarding students. Ma et al. tackle this complexity by integrating the Haddon matrix with interpretive structural modeling and analytic network process. They quantify risk factor weights across individual, family, community, and environmental levels, and find that risky behaviors and caregiver supervision together account for over 50% of influence. Sharif et al. add clinical intelligence, identifying admission laboratory markers and Glasgow Coma Scale scores as robust predictors of mortality and brain injury among pediatric near-drowning cases. These four papers span the two main currents in global drowning research: epidemiological work on risk perception, knowledge, and behavior, and clinical investigations of prognosis and severe outcome prediction (7).

## Road traffic injury: from child restraint misuse and disparity to emerging E-bike threats

Road traffic injury prevention receives close attention across several contexts. Kopańska et al. document widespread misuse of child restraint systems in Poland. Price and seat weight drive purchasing more than safety testing, and installation errors persist across all fixation types. Technical standards alone, the authors suggest, cannot bridge this evidence-practice chasm. Rose et al. analyze racial and ethnic disparities in motor vehicle crash fatalities among U.S. adolescents. American Indian and Alaska Native teens face mortality rates more than double those of White counterparts. In contrast, Black teen drivers had lower population-based fatality rates than White teen drivers. The author cautions that this apparent advantage may reflect differences in licensure, vehicle access, and driving exposure rather than genuine protective effect. Li Y. et al. apply fuzzy-set qualitative comparative analysis to e-bike injuries, identifying rigorous study design and behavioral risk analysis as core conditions for understanding severity in a mobility landscape where adolescents face heightened risks. These contributions speak to several pillars of the WHO's 10 strategies for keeping children safe on the road: appropriate child restraint use, managing adolescent driver risk, and addressing the expanding but poorly regulated domain of electric-powered two-wheelers (8).

## Beyond the dominant themes: falls, burns, poisoning, sports, intentional injury and injury nature

The Topic also reaches beyond drowning and road traffic injury to include causes that receive less attention in global rankings but carry serious burden. Xiong et al. use the Global Burden of Disease Study 2021 to quantify three decades of fall-related burden among Chinese children aged 0 to 14 years. They identify a post-2010 rebound in age-standardized incidence rates and a rising share of total disability-adjusted life-years (DALYs) accounted for by years lived with disability (YLDs)—signs that non-fatal sequelae are becoming a larger component of the overall burden, highlighting the need for sustained prevention and rehabilitation. Li T. et al. focus on an often-overlooked setting, reporting that medical care-related incidents and bed falls account for the majority of unintentional injuries among hospitalized children in western China. Toddlers aged 1 to 3 years comprise 45% of cases, and peak risk occurs during nursing shift changes. Chambon et al. characterize pediatric burn epidemiology in French Guiana, where scalding predominates and invasive infection disproportionately affects younger children with larger burned surface areas. Zheng et al. provide a 15-year retrospective on pediatric mushroom poisoning in Yunnan, identifying a pediatric-specific prognostic threshold distinct from adult threshold, and highlighting the vulnerability of school-aged children who forage with reduced supervision. Miao et al. map cross-sport patterns of health-related conditions among young Chinese athletes across 11 disciplines. Injuries among males concentrate in lower-extremity, while females show more diverse patterns involving upper extremities and trunk—findings that challenge one-size-fits-all prevention and call for sport- and sex-specific prevention strategies. Yang et al. document a 5-year escalating trend in emotional bullying among school-aged children in Jinan, with its prevalence rising from 10.4% to 14.4%. Teasing and social exclusion, rather than physical violence, drive the increase—a reminder that psychological injuries deserve equal epidemiological and preventive attention, especially in the post-pandemic context where academic pressure and limited social-emotional learning may be contributing factors.

Drowning and road traffic injuries consistently rank among the leading causes of child injury mortality in Global Burden of Disease analyses. Falls, burns, poisoning, sports injuries, and emotional bullying receive comparatively less attention in global rankings and research portfolios (2). Yet each imposes severe and often lifelong burdens on affected children, their families, and health systems, particularly in resource-limited settings where rehabilitation and mental health services are scarce. The inclusion of these less numerically represented but clinically and socially consequential injury types in this Research Topic is therefore particularly welcome. It makes visible a burden that mortality statistics alone cannot capture.

Two studies examine injury from the perspective of clinical nature and anatomical pattern. Bajuaifer et al. expose a striking knowledge deficit regarding pediatric concussion among Saudi parents: 63% report no prior awareness, and knowledge scores correlate positively with education and income, revealing a socioeconomic gradient in awareness of this increasingly recognized but still underappreciated injury type. Gu et al. apply multiple correspondence analysis and hierarchical clustering on principal components to identify five distinct patterns of multiple fractures in children, linking high-energy mechanisms to specific anatomical combinations.

## COVID-19 disruption: how population restrictions reshape injury patterns

The COVID-19 pandemic functioned as an unplanned natural experiment, and two studies use this disruption to show how restrictions reshape injury patterns in real time. Tang et al. analyze a decade of unintentional injury hospitalizations in Chongqing through interrupted time-series analysis. They demonstrate marked reductions during lockdown periods followed by gradual recovery, alongside a shift in age distribution toward older children and a significant increase in fall-related injuries post-pandemic. The authors attribute the post-pandemic rise to increased time spent in home environments combined with reduced structured supervision during school closures. Chen J. et al. examine product-related injuries in Xiamen through the lens of pandemic phases. Home-based injuries peaked during periods of non-pharmaceutical interventions (NPIs; e.g., school closures and stay-at-home orders) accounting for over 56% of all cases. More strikingly, injuries involving agro-forestry-fishery products were associated with markedly higher odds of severe injury during the NPI period—a pattern that the authors suggest may reflect population shifts from urban centers toward suburban or rural environments, exposing children to unfamiliar farming tools and more hazardous natural environments. These findings underline a point that injury prevention practitioners often forget: injury epidemiology is not static. It responds to social disruptions, policy shifts, migration patterns, and environmental changes. Surveillance systems should ideally detect such shifts as they emerge, while recognizing that many currently document them only retrospectively.

## Formative inquiry: understanding community readiness and implementation pathways

Before interventions can be scaled, formative research is needed to understand how communities perceive risk, mobilize existing resources, and navigate structural constraints. Bhatta et al. adopt a formative approach in rural Nepal, showing that embedding culturally tailored parental education within existing community health platforms offers a feasible pathway for home injury prevention in resource-limited settings. Two complementary qualitative studies from Guizhou, China by Li et al. (a) and Li et al. (b) examine how grassroots social organizations and other local stakeholders deliver innovative localized solutions, including grid-based management and round-the-clock patrols of high-risk areas, while also exposing knowledge distortion in secondary transmission training models.

## From evidence to action: interventions, innovations, and implementation

Knowing the burden is not enough. The test of public health research lies in translating evidence into interventions that are effective, scalable, and sustainable. Criel et al. update systematic review evidence for swimming and water safety training. Basic skills may reduce drowning mortality, and experiential pedagogies may outperform conventional instruction in improving aquatic competencies. The SwimSafe program in Bangladesh achieves remarkable cost-effectiveness at approximately 85 US dollars per DALY averted. Gao et al. report a community-based trial in Shanghai, showing that health education combined with WeChat engagement and AI-assisted voice follow-up significantly increased consistent child restraint system use and reduced orientation-related installation errors. The approach embeds digital health tools into routine community services such as vaccination clinics. Peters et al. provide experimental evidence that primary school children can master emergency calling after brief training. Dispatcher communication style exerts greater influence on call quality than instructional format—a detail that matters for training design. Chen Q. et al. report a cluster randomized controlled trial from rural Hebei, China. A comprehensive intervention integrating enhanced supervision, school-based health education, and environmental improvement achieved a crude reduction in injury incidence of 2.13 percentage points. Knowledge and protective behaviors improved substantially, though beliefs and risky behaviors did not, suggesting that deeper behavioral change may requires additional intervention strategies.

## Equity, vulnerability, and the structural determinants of safety

A thread that runs through these contributions is that injury risk is shaped by poverty, geography, governance gaps, and social exclusion. The harm falls heaviest on those with the least protection. Alam et al. show that geographic and socioeconomic disparities shape drowning risk perception in Bangladesh, with island residents reporting significantly lower perception scores and higher-income respondents reporting greater risk perception. Wang et al. identify children under grandparental care as a high-risk group in Henan, China. Supervision is present but often ineffective: caregivers were present in 54% of injury events, but effective supervision was lacking in 68% of these cases, and grandparental care conferred 33% higher injury odds compared to maternal care. Two studies from Guizhou (Li et al. (a), Li et al. (b)) illuminate a double concern in southwestern China—high environmental risk compounded by low institutional capacity. Resource constraints limit the reach of services to vulnerable children, while fragmented responsibilities, weak cross-sectoral coordination, and inadequate supervision can undermine the implementation of prevention efforts. Rose et al. highlight racial and ethnic inequities in traffic safety even in high-income settings and point to socioeconomic barriers as potential contributors, noting that differences in access to driver education, safer vehicles, licensure, and driving exposure may contribute to observed disparities. Yang et al. reveal that rural children experience higher social exclusion and threats, while urban children face elevated physical bullying—patterns that demand differentiated, context-specific strategies rather than uniform programs.

The message from these equity analyses is direct. Universal interventions will not close gaps on their own. They need to be accompanied by targeted strategies that address the social determinants of risk. The evidence supports differentiated approaches: school-based education for urban adolescents, community patrols for rural drowning hotspots, culturally adapted engagement for ethnic minorities, targeted support for vulnerable children, including left-behind children and children with disabilities, and equitable access to driver education and safer vehicles for disadvantaged teens.

## Gaps, limitations, and the path forward

Even with this breadth of evidence, several gaps stand out.

First, genuine child participation in injury prevention remains more aspirational than realized. Across the 26 contributions, children appear mainly as survey respondents or intervention recipients. Only a handful—notably the Guizhou qualitative work and Hebei's Child-To-Child approach—move toward meaningful engagement where children contribute to risk identification, peer education, and environmental auditing. The call for transformative child participation has not been fully answered. Future work should include participatory action research where children co-design interventions and evaluate outcomes.

Second, several priority injury types remain underrepresented. Animal-related injuries receive passing mention only. Climate disaster-related trauma, cyberbullying-linked self-harm beyond school-based measures, and suffocation warrant dedicated empirical attention. The absence of refugee and displaced child populations, and limited attention to children with disabilities, represent significant evidence gaps given their heightened vulnerability.

Third, data quality and surveillance infrastructure continue to constrain what analyses can reliably deliver. Facility-based studies capture only the tip of the iceberg. Community surveys rely on recall and proxy reporting. Administrative systems often lack the granularity needed for tracking mechanisms and long-term sequelae. Strengthening routine information systems, particularly in LMICs where the burden is greatest, is essential, as increasingly sophisticated analytic techniques cannot compensate for weak data generation.

## Conclusion

The 26 contributions show an increasingly integrative, methodologically diverse, and evidence-informed approach to child injury prevention. From three-decade mortality trend analyses to AI-assisted community interventions, from systematic reviews to formative qualitative research in remote villages, the field is producing increasingly precise intelligence about where, how, and among whom injuries occur. But precision is only a starting point. Sustainable transformation requires closing the implementation gap—turning evidence into enforced policies, funded programs, and outcomes that reach the last mile. Preventing child injury is integral to achieving multiple Sustainable Development Goals, including those related to child survival, road safety, and violence prevention, and requires recognizing injury not as an unfortunate accident, but as a structured, measurable and preventable public health problem with important implications for health equity. We hope this Research Topic captures current progress and spurs the cross-disciplinary, child-centered, equity-focused work that still lies ahead.
